# Electrophysiological evidence of different neural processing between visual and audiovisual inhibition of return

**DOI:** 10.1038/s41598-021-86999-1

**Published:** 2021-04-13

**Authors:** Xiaoyu Tang, Xueli Wang, Xing Peng, Qi Li, Chi Zhang, Aijun Wang, Ming Zhang

**Affiliations:** 1 School of Psychology, Liaoning Collaborative Innovation Center of Children and Adolescents Healthy Personality Assessment and Cultivation, Liaoning Normal University, Dalian, 116029 China; 2 Institute of Aviation Human Factors and Ergonomics, Civil Aviation Flight University of China, Guanghan, 618307 China; 3 School of Computer Science and Technology, Changchun University of Science and Technology, Changchun, 130022 China; 4 School of Biomedical Engineering, Faculty of Electronic Information and Electrical Engineering, Dalian University of Technology, Dalian, 116024 China; 5 Department of Psychology, Soochow University, Suzhou, 215123 China

**Keywords:** Neuroscience, Psychology

## Abstract

Inhibition of return (IOR) refers to the slower response to targets appearing on the same side as the cue (valid locations) than to targets appearing on the opposite side as the cue (invalid locations). Previous behaviour studies have found that the visual IOR is larger than the audiovisual IOR when focusing on both visual and auditory modalities. Utilising the high temporal resolution of the event-related potential (ERP) technique we explored the possible neural correlates with the behaviour IOR difference between visual and audiovisual targets. The behavioural results revealed that the visual IOR was larger than the audiovisual IOR. The ERP results showed that the visual IOR effect was generated from the P1 and N2 components, while the audiovisual IOR effect was derived only from the P3 component. Multisensory integration (MSI) of audiovisual targets occurred on the P1, N1 and P3 components, which may offset the reduced perceptual processing due to audiovisual IOR. The results of early and late differences in the neural processing of the visual IOR and audiovisual IOR imply that the two target types may have different inhibitory orientation mechanisms.

## Introduction

In a spatial cueing paradigm, the response to targets at the same side as the cue (also called the valid locations) is faster than that at the opposite side as the cue (invalid locations) when the cue and target stimulus onset asynchrony (SOA) is less than 300 ms. By contrast, when the SOA is more than 300 ms, the response to targets presented at valid locations is slower than that presenting at invalid locations. The latter phenomenon has been termed inhibition of return (IOR)^1^, and is described as an orientation mechanism to improve the efficiency of visual search by preventing the reinspection of already searched locations^2,3^.

IOR is observed within the visual^4^, auditory^5^, or tactile modalities^6^, suggesting that it may be a more general, than inhibitory process specific to unimodal search. Recently, researchers have extended the view to bimodal targets. Tang et al.^7^ investigated the IOR effect induced by visual, auditory and audiovisual targets. Their results showed a comparable IOR effect with audiovisual and visual targets when participants were asked to only focus on visual modality. However, they found that the magnitude of the IOR effect with bimodal audiovisual targets was smaller than that with unimodal visual targets when visual and auditory modalities were both attended (i.e., the divided attention condition). They also found that the simultaneous occurrence of visual and auditory targets was integrated (i.e., multisensory integration, MSI). The integrated bimodal stimuli can help improve signal salience^8^ and attract more attention^9,10^. The IOR actually reflect that attention is blocked from returning a previously attended location^11^ and facilitates visual exploration by reducing the perceptual salience of a previously explored location^12^. Thus, Tang et al. (2019) hypothesised that the integration of audiovisual targets enhances perceptual salience and outcompetes the reduced perceptual processing because of IOR, so that the IOR effect of audiovisual targets is decreased. No direct neural evidence has proven this hypothesis, although previous studies may indirectly support it.

On the one hand, multisensory integration and IOR seem to have common neural correlates. Tanaka et al.^13^ demonstrated that the superior colliculus (SC) has multisensory neurons responsive to visual, auditory, and tactile stimuli, but a suppressive interaction also exists in SC neurons. Macaluso and Driver^14^ showed that similar areas or even similar cells in subcortical areas or primary sensory cortices are responsible for both multisensory integration and crossmodal attention. Multisensory integration and attentional inhibition occur in similar brain areas, providing a possibility for spatial interplay between multisensory integration and IOR. On the other hand, multisensory integration can occur at multiple stages^15^. For example, early multisensory integration was found over the left parieto-occipital cortex (50-to-60 ms post-stimulus onset), sometimes followed by early positive modulation (80–100 ms) over occipital and temporal areas contralateral to audiovisual targets^16^. Multisensory integration also appeared at a time window of N2pc^17^ and even in later P3 stages^18,19^. Notably, many studies have found IOR during the abovementioned time windows^20–23^. The spatial and temporal overlaps of multisensory integration and IOR raise the possibility for their interaction at different levels. We hypothesised that at some time windows, particularly for some early ERP components, multisensory integration reduces IOR when these two effects appear simultaneously, or even eliminate IOR if MSI is sufficiently strong to compete with the decreased perceptual processing due to IOR.

Another more crucial question that must be considered is whether the time windows of IOR effects for unimodal visual targets and bimodal audiovisual targets are different due to the influence of multisensory integration or physical factors. Previous ERP studies have shown that the suppression of perceptual processing in extrastriate visual areas is the most common inhibition associated with the visual IOR effect—namely, a reduced amplitude of the earlier P1/N1 component at the valid locations compared with that at the invalid locations^22,24–26^. IOR is also linked to the visual N2 component with a frontal/central^27,28^ or a posterior scalp distribution^29^ involved in inhibitory executive processes—similarly, the amplitude is reduced at the valid locations^30,31^. Additionally, a significant IOR effect was reported occasionally in association with a special P3 enhancements for valid locations compared with that for invalid locations^6,23^, although this effect has not very often been reported^19,24,32^. In different tasks, these ERP components may contribute to the visual IOR separately or collectively, and corresponding theories involve early perceptual inhibition^25,33,34^, late motor inhibition^35,36^ or dual-component interpretation^21,37–39^.

The neural activities of audiovisual stimuli are not equal to the neural activities of auditory or visual stimuli at some stages^40^. P3 reflects the updating of working memory during the postdetection process^41^, it was viewed as an index of context updating^42^ with larger amplitudes elicited by an increased amount of engaged attentional resources and/or processing capacity^43^. Audiovisual targets are novel stimuli compared with previous visual cues, which can attract more attention^44^ or may lead to updated working memory after these target stimuli are detected. Visual targets have no modality novelty relative to visual cues, and the IOR is mainly related to the inhibition in early visual perception^45^. The audiovisual targets might elicit a late IOR effect associated with the P3 component, and significant P1, and N1 inhibitory components should be found in visual targets. Because multisensory integration is more likely to occur in the early processing^46–48^ stages, it may cause the early P1 and N1 to be reduced or even disappear in audiovisual targets. Clarifying the problem of time windows may contribute to a better understanding of the behaviour difference between the auditory and visual IOR.

The primary goal of this study was to examine whether the enhanced perceptual salience induced by multisensory integration is the cause of the audiovisual IOR being smaller than the visual IOR. Considering that behavioural indicators (reaction time and accuracy) of the IOR effect usually reflect the sum of all information processing stages, the processing differences between visual IOR and audiovisual IOR cannot be unequivocally clarified from the internal mechanism. Therefore, we adopted a cue-target paradigm, using event-related potential (ERP) technique in the present study, to examine the neural correlates with the behaviour IOR differences between target types. In this paradigm, a thickened white box appears on the left or right side of the fixation as an exogenous cue that triggers exogenous spatial orientation. To facilitate the occurrence of an IOR, a central reorientation method is used, with the previous fixation extended to focus attention back to the centre. We manipulated the location validity (valid and invalid), and stimuli type (visual, auditory, and audiovisual stimuli) and asked participants to focus on both auditory and visual information simultaneously and then perform a target/non-target discrimination task (responding to the target, and withholding responding to the non-target). Finally, we compared the accuracy (ACC), mean reaction time (RT) or ERP amplitude of the target stimuli at the valid locations with those at the invalid locations. As explained earlier, we hypothesised that the enhanced perceptual salience induced by multisensory integration outcompetes the reduced perceptual processing partially because of IOR, leading to a decrease in the early IOR effect on audiovisual targets, that will be reflected in neural processes.

## Method

### Participants

We calculated the appropriate sample size based on the G*Power toolbox^49^ and work of Van der Stoep^50^. To achieve the recommended 95% statistical power at *α* = 0.05, and an effect size of 0.70, the appropriate sample size was defined as at least 12 participants. Twenty undergraduate students (mean age = 20.33 years; SD = 2; 17 women, 3 men; 1 left-handed) were eventually recruited to participate in the current study. All the students had normal or corrected-to-normal vision and normal hearing capabilities, and had no neurological or psychiatric disorders. This study was approved by the Ethics Committee of Liaoning Normal University and was performed in accordance with the approved guidelines and the Declaration of Helsinki. All the participants provided written informed consent before participating.

### Stimuli

**Figure 1:**
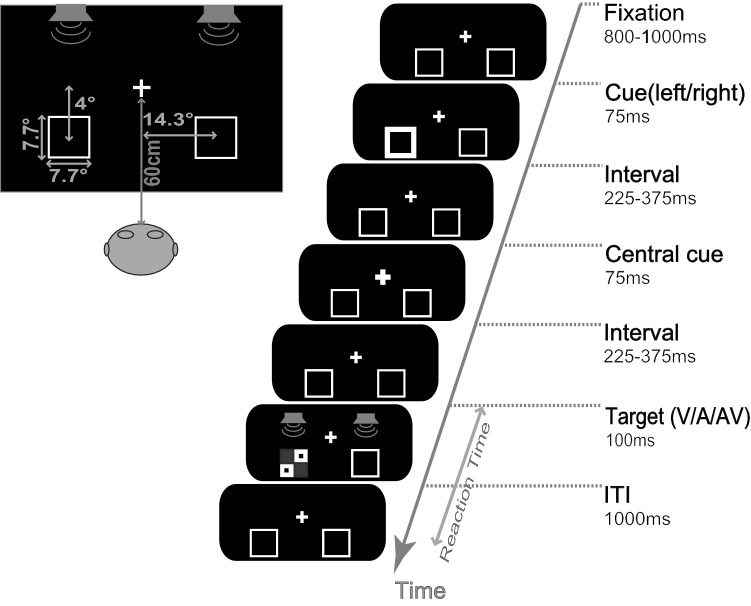
Illustration of the stimuli and experimental procedure. The size and position of the stimuli are shown in the left panel. The sequence of events and their duration (starting from the top) in the valid location of the visual (V) target condition are illustrated in the right panel.

The experiment was conducted in a dimly illuminated, sound-attenuated room. The participants were seated directly in front of a computer monitor, with their heads held steady by a chin rest. All visual (V) stimuli were presented on a black background display (with a screen resolution of 1024 × 768 black and a refresh rate of 100 Hz) 60 cm from the participant (see Fig. 1). The visual target was a checkerboard with two dots, and the visual non-target was a checkerboard without dots. Auditory (A) stimuli were presented via two speakers placed at both the left rear and right rear of the display. White noise was set as the auditory target (65 dB, rise/fall times of 10 ms) and another pure tone at 1600 Hz (65 dB, rise/fall times of 10 ms) was set as auditory non-target. Audiovisual (AV) stimulus comprised a combination of the simultaneous presentation of both auditory and visual target/non-target. For example, a checkerboard with two dots and white noise comprised an audiovisual target, a checkerboard without two dots and a pure tone comprised an audiovisual non-target. The fixation point (0.05° × 0.05° of the visual angle) was a small white cross (RGB, 255, 255, 255; 155.2 cd/m^2^) flanked by two white square outline boxes (7.7° × 7.7°), presented 4° below and 14.3° to the left and right of the fixation across.

### Procedure and task

The experimental procedure is illustrated in Fig. 1. Each trial started with a fixation stimulus that appeared for 800–1000 ms. Subsequently, one of the white square boxes became thicker as a peripheral visual cue, which lasted for 75 ms. After a random first interval of 225–375 ms, the fixation cross was extended to 0.1° × 0.1° to serve as a reorienting cue (lasting 75 ms) to bring focused attention back to the centre. Next, the second interval randomly appeared again for 225–375 ms. Thus, the SOAs were randomly assigned between 600 and 900 ms to help resist the temporal expectation of the appearance of the targets. Next, the A, V or AV targets/non-targets were randomly presented for 100 ms on the left/right side. Finally, the fixation stimulus appeared for 1000 ms to allow the participants to make reaction, after which the next trial started automatically. The participants were required to focus on both the visual and auditory modalities simultaneously. They performed a discrimination task in which they were requested to judge whether the stimulus was a target or a non-target. The participants were instructed to press "B" on the keyboard as quickly and accurately as possible when they detected an auditory, a visual, or an audiovisual target, but withhold their response when a non-target stimulus appeared in the left or right location. During the experiment, the participants were instructed to reduce their actions of swallowing, frowning, and blinking and to, keep their bodies from moving. Matters requiring attention were only target stimuli trials that were used in further analyses.

### Design

Two factors of location validity and target type were manipulated in the behaviour experiment. Location validity involved two levels: valid and invalid. Half of the trials in each block involved valid locations, and half involved invalid locations. There were three target types: A targets, V targets and AV targets. Before the formal experiment, each participant needed to complete 60 practice trials first to ensure that the instructions were clear and that the task was performed correctly. The formal experiment comprised 10 blocks of 1200 trials: 960 target trials (80%) and 240 non-target trials (20%). There were 6 experimental conditions and 200 trials contained for each; 160 were target trials, and 40 were non-target trials. All the conditions contained an equal number of A, V, and AV stimuli trials. Within each block, the participants were asked to respond to the targets, and their EEG data were recorded simultaneously. Feedback about the number of correct responses was provided at the end of each block, and rest periods were provided between blocks. The order in which the participants completed the target condition blocks was randomised and counterbalanced across the participants.

### Behavioural data recording and analysis

E-prime software (1.0 version) was used to present the stimuli and record the responses. Trials with RTs faster than 100 ms (anticipations) or slower than 1000 ms (misses) were counted as errors. These error trials and trials with incorrect responses were excluded from analysis. Thus, 2% of the target trials were removed from further analysis. The mean reaction time (RT) and accuracy (ACC) were entered into a 3 (target type: A, V, AV) × 2 (location validity: valid and invalid) repeated measures ANOVA. Paired *t* test (two-tailed) was used to compare the IOR effect (valid vs*.* invalid) in different target modalities. All statistical significance levels were set at 0.05. The effect size of Cohen’s *d* or partial eta-squared (*η*_*p*_^2^) was calculated for mean comparisons or ANOVA, respectively.

### ERP data recording and analysis

An EEG system (Brain Products, Brain Vision Recorder 2.0) was used to record EEG signals using 32 electrodes mounted on an electrode cap as specified by the International 10–20 system, with the reference electrode on the left earlobe and a ground electrode on the medial frontal aspect. A horizontal electrooculogram (EOG) was recorded from the outer canthi of the left eye, and a vertical EOG was recorded from an electrode placed 1.5 cm above and below the left eye. The raw signals were digitised with a sample frequency of 500 Hz, and all impedances were kept below 5 kΩ.

Offline, an EEG system (Brain Products, Brain Vision Analyzer 2.0) was used to analyse ERP signals. The data were digitally filtered with a high-pass filter of 0.1 Hz and a low-pass filter of 30 Hz (24 dB/octave)^51^. The data were segmented into 1000 ms epochs from − 200 to 800 ms. Artefact rejection was performed using a semi-automated procedure^52^ to remove epochs that contained eye movements and blinks from EEG. Also, trials with excessive artefacts (± 80 μV) were excluded from further analysis. Finally, the data from each electrode were averaged and a grand average ERP was computed across all participants for each experimental condition.

According to previous studies, the present ERP components were divided by the time windows in which they occurred: P1, 90–140 ms; N1, 150–190 ms; N2, 220–290 ms; P3, 350–400 ms. We selected electrodes P3, P4, P7, P8, CP5, CP6, F3, F4, F7, F8, FC5, FC6, and Cz^53^ in the lateral and frontal, central, and occipital areas for statistical analyses of P1, N1, N2, and P3. In each time window, the mean amplitudes were analysed using repeated measures ANOVA with factors of location validity and electrode.

## Results

### Behavioral results

Accuracy rate analysis showed that only the main effect of the target type was significant [*F*(2, 38) = 7.328; *p* *<* 0.01; *η*_*p*_^2^ = 0.278]. The response to the AV targets (97%) was more accurate than those to the A targets (95%) and V targets (95%). No other significant main effects or interactions were found. For the mean correct RTs, ANOVA revealed a significant main effect of the target type [*F*(2, 38) = 29.194; *p* < 0.001; *η*_*p*_^2^ = 0.606], and the RTs to AV targets (408 ms) were significantly shorter than those to V (468 ms) and A targets (453 ms). The main effect of the location validity was significant [*F*(1, 19) = 45.747, *p* < 0.001, *η*_*p*_^2^ = 0.707], reflecting faster RTs at the invalid locations (437 ms) than at the valid locations (448 ms). Crucially, the interaction between the target type and location validity was significant [*F*(2, 38) = 18.828; *p* < 0.001; *η*_*p*_^2^ = 0.498]. The difference in RTs between valid and invalid locations was significant for the V targets [*t* (19) = 10.23; *p* < 0.01; *d* = 0.360] and the AV targets [*t* (19) = 7.04; *p* < 0.001; *d* = 0.164], but not for the A targets [*t* (19) = 0.49; *p* = 0.628; *d* = 0.028]. Further analysis found that the IOR effect of the V targets (21 ms) was larger than that of the AV targets (10 ms) [*t* (19) = − 5.75; *p* < 0.001; *d* = 1.362].

### ERP results

Consistent with the behavioural results of previous studies, no auditory IOR was found. We still observed that the visual and audiovisual IOR effects were different in magnitude. The reasons for the differences are not clear at present. Thus, we provide a neural process comparison across the A targets, V targets and AV targets. The mean amplitudes were subjected to a 2 (location validity: valid and invalid) × 13 (electrode) two factor repeated measures ANOVA.

### Event-related potential of the auditory IOR effect: (A valid vs. A invalid)

#### P1 component (90–140 ms)

**Figure 2:**
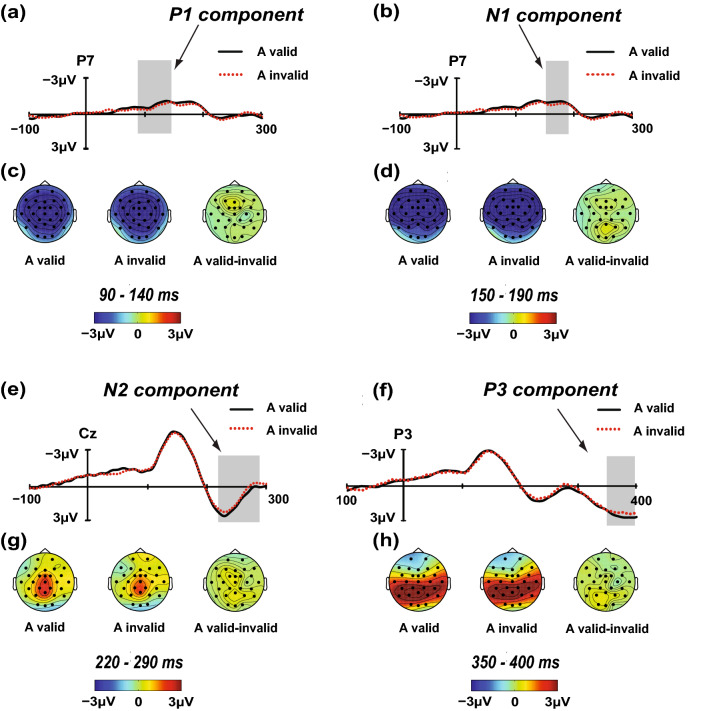
Direct comparisons of the auditory IOR effect for the time windows 90–140 ms, 150–190 ms, 220–290 ms, and 350–400 ms. The contrasts between the valid and invalid locations were analysed using *t* test (sig. two-tailed; 95% confidence interval, CI), and the maximum difference between the valid and invalid locations is shown from P7 electrode, P7 electrode, Cz electrode and P3 electrode, for the P1, N1, N2 and P3 components, respectively. The grand average amplitudes elicited by the A targets presented in either the valid (black solid) or invalid (red dotted) locations are shown in panels (**a**), (**b**), (**e**), and (**f**). The scalp topographies of the P1, N1, N2 and P3 components in the valid and invalid waveforms are shown in panels (**c**), (**d**), (**g**), and (**h**), respectively. Valid-invalid scalp topographies represent the IOR effect.

Figure 2a,c shows the P1 waveforms and scalp topographies of the A targets, respectively. The analysis yielded a significant main effect for the electrode [*F*(12, 228) = 11.480; *p* < 0.001; *η*_*p*_^2^ = 0.377]. Not surprisingly, neither the main effect of the location validity [*F*(1, 19) = 0.062; *p* = 0.806; *η*_*p*_^2^ = 0.003], nor the interaction between the location validity and electrode [*F*(12, 228) = 0.437; *p* = 0.947; *η*_*p*_^2^ = 0.022] were significant. The maximum difference between the valid and invalid locations was found in the P7 electrode (P7v-i = − 0.273 μV;* p* = 0.476; v-i means valid minus invalid).

#### N1 component (150–190 ms)

Figure 2b,d shows the N1 waveforms and scalp topographies of the A targets. The main effect of the electrodes [*F*(12, 228) = 11.380; *p* < 0.001; *η*_*p*_^2^ = 0.431] was significant. Similarly, neither the main effect of the location validity [*F*(1, 19) = 0.065; *p* = 0.802; *η*_*p*_^2^ = 0.003], nor the interaction between the location validity and electrode [*F*(12, 228) = 0.378; *p* = 0.970; *η*_*p*_^2^ = 0.019] was significant. The maximum difference between the valid and invalid locations was found in the P7 electrode (P7v-i = − 0.288 μV;* p* = 0.331).

#### N2 component (220–290 ms)

Figure 2e,g shows the waveforms and scalp topographies of the N2 components elicited by the A targets. The main effect of the electrode [*F*(12, 228) = 3.399; *p* < 0.001; *η*_*p*_^2^ = 0.152] was observed, but the main effect of the location validity [*F*(1, 19) = 0.602; *p* = 0.447; *η*_*p*_^2^ = 0.031] was not observed. No interaction was found between the location validity and electrode [*F*(12, 228) = 0.697; *p* = 0.754; *η*_*p*_^2^ = 0.035]. The maximum difference between the valid and invalid locations was found in the Cz electrode (Cz v-i = 0.521 μV;* p* = 0.253).

#### P3 component (350–400 ms)

The P3 waveforms and scalp topographies of the A targets are included in Fig. 2f,h. The analysis yielded a significant main effect for the electrode [*F*(12, 228) = 17.469; *p* < 0.001; *η*_*p*_^2^ = 0.479]. We did not find a significant effect of the location validity [*F*(1, 19) = 0.205; *p* = 0.656; *η*_*p*_^2^ = 0.011] and a significant interaction between the location validity and electrode [*F*(12, 228) = 0.631; *p* = 0.815; *η*_*p*_^2^ = 0.032]. The maximum difference between the valid and invalid locations was found in the P3 electrode (P3v-i = 0.488 μV;* p* = 0.247).

### Event-related potential of the visual IOR effect: (V valid vs. V invalid)

#### P1 component (90–140 ms)

**Figure 3:**
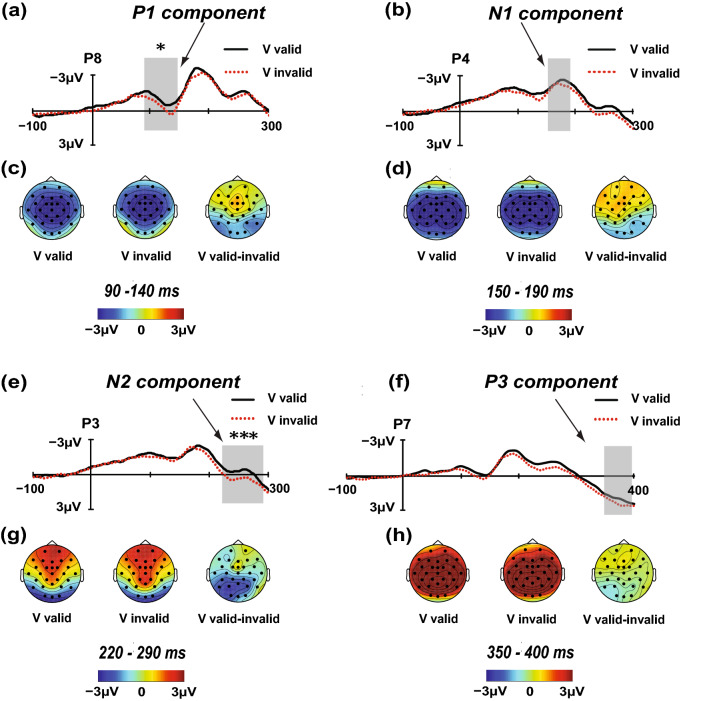
Direct comparisons of the visual IOR effect for the time windows 90–140 ms, 150–190 ms, 220–290 ms, and 350–400 ms. The contrasts between the valid and invalid locations were analysed using *t* test (sig. two-tailed; 95% confidence interval, CI), and the maximum difference between the valid and invalid locations is shown from P4 electrode, and P7 electrode, for the N1 and P3 components, respectively. The difference between the valid and invalid locations for the P1 and N2 components reaches significance in the P8 electrode and P3 electrode, respectively (*p < 0.05; ***p < 0.001). The grand average amplitudes elicited by the V targets presented in either the valid (black solid) or invalid (red dotted) locations are shown from panels (**a**), (**b**), (**e**), and (**f**). The scalp topographies of the P1, N1, N2 and P3 components in the valid and invalid waveforms are shown in panels (**c**), (**d**), (**g**), and (**h**), respectively. Valid-invalid scalp topographies represent the IOR effect.

Figure 3a,c shows the P1 waveforms and scalp topographies of the V targets, respectively. There was a main effect of the electrode [*F*(12, 228) = 11.865; *p* < 0.001; *η*_*p*_^2^ = 0.384] but no significant main effect of the location validity [*F*(1, 19) = 0.302; *p* = 0.589; *η*_*p*_^2^ = 0.016]. Importantly, the interaction between the location validity and electrode was significant [*F*(12, 228) = 2.414; *p* < 0.01; *η*_*p*_^2^ = 0.113]. Simple effect analysis showed that significant or marginally significant differences between the valid and invalid locations occurred at electrodes P4, P7, and P8 (P4v-i = − 0.507 μV, *p* = 0.06; P7v-i = − 0.506 μV, *p* < 0.01; P8v-i = − 0.538 μV, *p* < 0.05), and a significant maximum difference between the valid and invalid locations was found in the P8 electrode (− 0.538 μV).

#### N1 component (150–190 ms)

Figure 3b,d shows the N1 waveforms and scalp topographies of the V targets. The main effect of the location validity [*F*(1, 19) = 0.076; *p* = 0.786; *η*_*p*_^2^ = 0.004] was not significant. However, the main effect of the electrode [*F*(12, 228) = 3.723; *p* < 0.001; *η*_*p*_^2^ = 0.164] was significant. The location validity also interacted with electrode [*F*(12, 228) = 2.029; *p* < 0.05; *η*_*p*_^2^ = 0.096]. Further simple effect analysis revealed no significant differences between the valid and invalid locations at any of the 13 electrodes. Only a maximum difference between the valid and invalid locations was found in the P4 electrode (P4v-i = − 0.386 μV;* p* = 0.194).

#### N2 component (220–290 ms)

The N2 waveforms and scalp topographies of the V targets are illustrated in Fig. 3e,g. ANOVA revealed a significant main effect of the electrode [*F*(12, 228) = 13.421; *p* < 0.001; *η*_*p*_^2^ = 0.414]. ANOVA also revealed significant interaction between the location validity and electrode [*F*(12, 228) = 1.805; *p* < 0.05; *η*_*p*_^2^ = 0.087]. No main effect of the location validity was obtained [*F*(1, 19) = 1.965; *p* = 0.177; *η*_*p*_^2^ = 0.094]. Simple effect analysis revealed significant amplitude differences between the valid and invalid locations at electrodes P3, P4, P7, and CP5 (P3v-i = − 0.880 μV, *p* < 0.001; P4v-i = − 0.730 μV, *p* < 0.05; P7v-i = − 0.692 μV, *p* < 0.01; CP5v-i = − 0.599 μV, *p* < 0.01). The significant maximum difference was found in the P3 electrode (− 0.880 μV) (Supplementary Information).

#### P3 component (350–400 ms)

The P3 waveforms and scalp topographies of the V targets are displayed in Fig. 3f,h. No main effect of the location validity was observed [*F*(1, 19) = 0.001; *p* = 0.979; *η*_*p*_^2^ = 0.000]. A significant main effect of the electrode was found [*F*(12, 228) = 8.615; *p* < 0.001; *η*_*p*_^2^ = 0.312], and a significant interaction between the location validity and electrode was also observed [*F*(12, 228) = 2.599; *p* < 0.01; *η*_*p*_^2^ = 0.120]. However, simple effect analysis revealed no significant differences between the valid and invalid locations at any of the 13 electrodes. The maximum difference was found in the P7 electrode (P7v-i = − 0.567 μV;* p* = 0.072).

### Event-related potential of the audiovisual IOR effect: (AV valid vs. AV invalid)

#### P1 component (90–140 ms)

**Figure 4:**
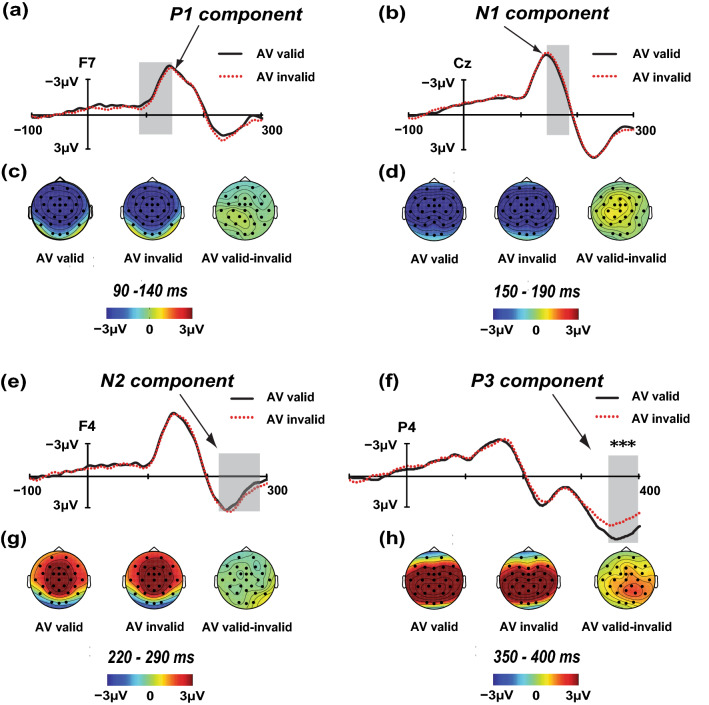
Direct comparisons of the audiovisual IOR effect for the time windows 90–140 ms, 150–190 ms, 220–290 ms, and 350–400 ms. The contrasts between the valid and invalid locations were analysed using *t* test (sig. two-tailed; 95% confidence interval, CI), and the maximum difference between the valid and invalid locations is shown from F7 electrode, Cz electrode, and F4 electrode, for the P1, N1 and N2 components, respectively. The difference between the valid and invalid locations for the P3 component reaches significance in the P4 electrode (***p < 0.001). The grand average amplitudes elicited by the AV targets presented in either the valid (black solid) or invalid (red dotted) locations are shown in panels (**a**), (**b**), (**e**), and (**f**). The scalp topographies of the P1, N1, N2 and P3 components in the valid and invalid waveforms are shown in panels (**c**), (**d**), (**g**), and (**h**), respectively. Valid-invalid scalp topographies represent the IOR effect.

Figure 4a,c shows the P1 waveforms and scalp topographies of the AV targets, respectively. We observed a significant main effect of the electrode [*F*(12, 228) = 20.112; *p* < 0.001; *η*_*p*_^2^ = 0.514]. The main effect of the location validity [*F*(1, 19) = 0.321; *p* = 0.577; *η*_*p*_^2^ = 0.017], and interaction between the location validity and electrode were not significant [*F*(12, 228) = 0.787; *p* = 0.663; *η*_*p*_^2^ = 0.040]. The maximum difference between the valid and invalid locations was found in the F7 electrode (F7v-i = − 0.393 μV; *p* = 0.110).

#### N1 component (150–190 ms)

Figure 4b,d shows the waveforms and scalp topographies of the N1 components elicited by the AV targets. The main effect of the electrode [*F*(12, 228) = 10.393; *p* < 0.001; *η*_*p*_^2^ = 0.354] was significant. The main effect of the location validity [*F*(1, 19) = 1.141; *p* = 0.299; *η*_*p*_^2^ = 0.057] and interaction between the location validity and electrode [*F*(12, 228) = 0.480; *p* = 0.925; *η*_*p*_^2^ = 0.025] were not significant. The maximum difference between the valid and invalid locations was found in the Cz electrode (Cz v-i = 0.506 μV;* p* = 0.255).

#### N2 component (220–290 ms)

As shown in Fig. 4e,g, the analysis of the N2 component showed a significant main effect of the electrode [*F*(12, 228) = 8.373; *p* < 0.001; *η*_*p*_^2^ = 0.306], while no significant main effect of the location validity [*F*(1, 19) = 0.814; *p* = 0.378; *η*_*p*_^2^ = 0.041] or a significant interaction between the location validity and electrode [*F*(12, 228) = 1.113; *p* = 0.351; *η*_*p*_^2^ = 0.055] was found. The maximum difference between the valid and invalid locations was found in the F4 electrode (F4v-i = − 0.529 μV;* p* = 0.260).

#### P3 component (350–400 ms)

The P3 waveforms and scalp topographies of the AV targets are illustrated in Fig. 4f,h. We observed a significant main effect of the location validity [*F*(1, 19) = 19.338; *p* < 0.001; *η*_*p*_^2^ = 0.504], and a significant main effect of the electrode [*F*(12, 228) = 14.631; *p* < 0.001; *η*_*p*_^2^ = 0.435]. We also found a significant interaction between the location validity and electrode [*F*(12, 228) = 4.381; *p* < 0.001; *η*_*p*_^2^ = 0.187]. As in a previous study, a subsequent simple analysis revealed a larger P3 amplitude in the valid location for the AV targets, the involved electrodes were CP5, P3, P7, P4, P8, CP6, Cz, FC6, and F8 (CP5v-i = 1.032 μV, *p* < 0.01; P3v-i = 1.272 μV, *p* < 0.01; P7v-i = 0.680 μV, *p* < 0.05; P4v-i = 1.892 μV, *p* < 0.001; P8v-i = 1.406 μV, *p* < 0.001; CP6v-i = 1.472 μV, *p* < 0.001; CZv-i = 1.222 μV, *p* < 0.01; FC6v-i = 0.739 μV, *p* < 0.05; F8v-i = 0.643 μV, *p* < 0.05). A significant maximum difference was found in the P4 electrode (1.892 μV).

### MSI effect (AV vs. A + V)

**Figure 5:**
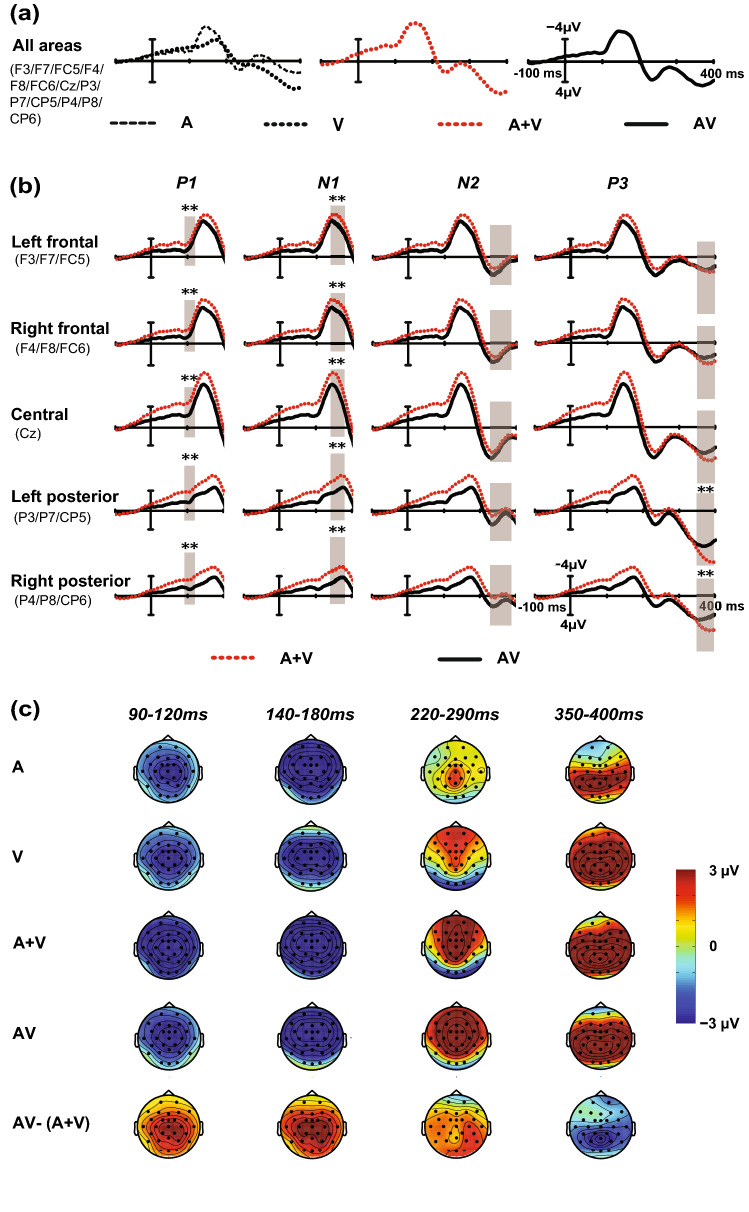
Multisensory integration effect for the AV targets. Panel (**a**) displays the mean ERP waveforms of 13 electrodes to A (black broken), V (black dotted), A + V (red dotted) and AV (black solid). The five regions of ERP waves in panel (**b**) depicts the distribution of the multisensory integration effect in four windows, shown separately for the 90–120 ms, 140–180 ms, 220–290 ms, and 350–400 ms windows. Significant difference between the bimodal AV (black solid) and summed unimodal A + V (red dotted) ERP waveforms (sig. two-tailed; 95% confidence interval, CI), reflecting a multisensory integration effect for the AV targets and marked by the symbol ‘**’ (*p* < 0.01). Scalp topographies of unimodal A, unimodal V, summed unimodal A + V, and bimodal AV and the AV-(A + V) processing are depicted in panel (**c**).

According to the above results, the difference in the time course between the visual IOR and audiovisual IOR mainly exists for the P1, N2 and P3 components. The visual IOR was generated from the first two stages, while the audiovisual IOR appeared in only one late processing stage. In this study, we employed a bimodal divided attention task, which may be a key factor. Multisensory integration could occur when targets from both the visual and auditory modalities are presented simultaneously. According to a previous study, multisensory integration played an essential role in partially offsetting the audiovisual IOR effect. This effect was demonstrated by comparing the ERP data of the AV stimuli with the sum of the ERP to the corresponding A and V stimuli, and showing differences between the AV and A + V waveforms^54^. To fully elucidate the reasons for the time course differences between the two IOR effects, the multisensory integration effect was analysed at the time windows of P1 (90–120 ms), N1 (140–180 ms), N2 (220–290 ms) and P3 (350–400 ms) in the corresponding analysis window of the IOR. Figure 5a shows the mean ERP waveforms of 13 electrodes to A, V, A + V and AV targets. To better understand the brain regions where integration occurs, Fig. 5b shows the mean ERP waveforms of 5 cortical regions to AV and A + V. The corresponding scalp topographies of A, V, A + V, AV and AV − (A + V) are shown in Fig. 5c. An overall ANOVA contained the following within-subject factors: target type (2: AV or A + V) and cortical region (5 levels: left frontal F3/F7/FC5, right frontal F4/F8/FC6, central Cz, left posterior P3/P7/CP5 and right posterior P4/P8/CP6).

#### P1 component (90–120 ms)

The test for the P1 component indicated that the main effects of the target type [*F*(1, 19) = 34.819; *p* < 0.001; *η*_*p*_^2^ = 0.647] and cortical region [*F*(4, 76) = 9.512; *p* < 0.001; *η*_*p*_^2^ = 0.334] were significant. The P1 amplitude elicited by the AV targets (− 2.535 μV) was larger than the sum (− 4.495 μV) of the P1 amplitudes elicited by the A targets and V targets. A statistically significant interaction [*F*(4, 76) = 7.767; *p* < 0.001; *η*_*p*_^2^ = 0.29] between target type and cortical region revealed that the P1 amplitude was reduced for the A + V targets relative to AV targets over all five regions (*p* < 0.01).

#### N1 component (140–180 ms)

For the N1 amplitude, the analysis yielded a main effect of the target type [*F*(1, 19) = 29.157; *p* < 0.001; *η*_*p*_^2^ = 0.605] and cortical region [*F*(4, 76) = 21.221; *p* < 0.001; *η*_*p*_^2^ = 0.528], and the amplitude of the AV targets (− 5.767 μV) was smaller than the sum of the amplitudes of A + V (− 8.154 μV). A significant interaction was also found between the target type and cortical region [*F*(4, 76) = 4.634; *p* < 0.01; *η*_*p*_^2^ = 0.196], and simple effect analysis revealed a multisensory integration effect in all five regions (*p* < 0.01).

#### N2 component (220–290 ms)

Similarly, the results revealed that both the target type [*F*(1, 19) = 7.246; *p* < 0.05; *η*_*p*_^2^ = 0.335] and cortical region [*F*(4, 76) = 9.581; *p* < 0.001; *η*_*p*_^2^ = 0.335] were significant, with smaller negative amplitudes of the AV targets (2.576 μV) than the sum of the amplitudes of A + V (1.444 μV). However, the interaction between the target type and cortical region was not significant [*F*(4, 76) = 1.191; *p* = 0.322; *η*_*p*_^2^ = 0.059].

#### P3 component (350–400 ms)

The P3 amplitude was expressed in either a main effect of the target type [*F*(1, 19) = 5.267; *p* < 0.05; *η*_*p*_^2^ = 0.217] or cortical region [*F*(4, 76) = 10.852; *p* < 0.001; *η*_*p*_^2^ = 0.364], with a stronger P3 amplitude in the A + V (6.243 μV) targets than in the AV targets (4.549 μV). Additionally, the two-way interaction between the target type and electrode was significant [*F*(4, 76) = 4.244; *p* < 0.01; *η*_*p*_^2^ = 0.183], resulting from a greater positive amplitude on the A + V waveforms than on the AV waveform at the left posterior region (*p* < 0.01) and right posterior region (*p* < 0.01).

In summary, multisensory integration emerged in three processing stages: the earliest integration appeared in all five cortical areas and over 90–120 ms. The second integration appeared at 140–180 ms and covered these five areas, and the last integration mainly occurred in the left posterior region and the right posterior region for the time window of 350–400 ms.

## Discussion

This study aimed to examine the electrophysiological underpinning of behaviour IOR differences between visual targets and audiovisual targets. Our behavioural results confirmed previous studies^7^ in which the IOR effect of the visual targets was larger than that of the audiovisual targets when participants focused on both the visual and auditory modalities, while no auditory IOR effect was observed. By comparing the ERP results of the auditory targets, visual targets and audiovisual targets, the visual IOR appeared at two processing stages (P1, N2), while only the late stage (P3) had a significant audiovisual IOR. The ERP results suggested that the time courses of visual IOR and audiovisual IOR were different.

As expected, we found no auditory IOR at the four processing stages. Although the existence of the auditory IOR has now been demonstrated, little is known about the factors that might limit this effect. However, much more is known about the factors that limit the visual IOR^55^. The visual lOR has usually been attributed to the operation of an exogenous (involuntary) attentional mechanism. Rafal et al.^56,57^ showed that the visual IOR is eliminated when the likelihood that the cue provides accurate spatial information is better than chance. This happens because the predictability of spatial relations may activate the endogenous (voluntary) orienting mechanism, while the higher-level endogenous orienting mechanism system appears to be able to modify the effects of the lower-level exogenous attentional mechanism^58^. By contrast, the results of Mondor's^55^ study showed that predictability substantially influences the time course of the inhibitory component of the auditory IOR. An auditory IOR effect became apparent at a longer SOA when either the temporal or the spatial relationship was predictable, rather than when either was unpredictable. The auditory and visual inhibitory seems to have different bases for selective attention. In this study, we used a cue–target paradigm to examine the effect of uninformative visual cues on IOR effects. Moreover, we only set a single SOA that was randomly presented in 600–900 ms. According to Mondor's views, either the temporal or spatial relationship is unpredictable, which might be the key factors in failure to identify the auditory IOR. Coincidentally, Prime and Word's^59^ work demonstrates that such auditory IOR effects are not found in the target–target paradigm or cue–target paradigm requiring Go/NoGo discrimination of the targets. The latter is the paradigm used in current experiments. Not surprisingly, the auditory IOR was missed. We were interested in revealing the neural processing truth of the visual IOR and audiovisual IOR of different magnitudes.

In our study, we found no early P1 neural modulation for the audiovisual IOR, likely because of multisensory integration. There is converging evidence to suggest that it leads to multisensory integration when auditory and visual stimuli are simultaneously presented^60^. More importantly, multisensory integration could affect IOR components elicited by exogenous cues^61^. Combined with our studies, the audiovisual stimuli were a combination of auditory and visual stimuli presented simultaneously, indicating that multisensory integration could occur. To illustrate the effect of multisensory integration on IOR, we analysed the time course of the multisensory integration of audiovisual targets. As expected, significant differences between the ERP amplitude of AV and combined ERP amplitude of A + V were found in the three time windows of P1, N1 and P3, i.e., multisensory integration occurred at multiple stages of processing. Previous studies have confirmed that multisensory integration more frequently appeared at the P50 stage but also existed at a relatively late stage of processing^16,48^. We found early multisensory integration in all five cortical regions, and this strong integration indeed caused the absence of an early audiovisual IOR in all brain regions. Only the left posterior region and right posterior region induced the late multisensory integration effect, which cannot offset the IOR effect in the right frontal region (F8/FC6) or central region (Cz), resulting in the partial retention of the late audiovisual IOR. Instead, the unimodal targets do not have the conditions for multisensory integration, and the visual IOR effect can appear on the relatively early P1 and N2 components. The current results confirm the hypothesis that the power of enhanced perceptual salience by audiovisual integration offsets the reduced perceptual processing due to IOR, but not in every stage. When the IOR (decreasing salience) encounters the integration of attended visual and attended auditory stimuli (increasing perceptual salience), a reduced IOR effect elicited by an audiovisual target is found. Taken together, multisensory integration of the audiovisual targets provides some explanations for the magnitude and time course differences between the visual IOR and audiovisual IOR.

However, multisensory integration does not fully explain the time course differences between the visual IOR and the audiovisual IOR. First, audiovisual targets have neither MSI nor the IOR associated with the N2 component, which is a more reliable neural marker of IOR^30^. For the late response-related N2 component, the IOR was affected by the proportion of Go/NoGo stimuli. The N2 effects were reduced with the decreasing probability of Go stimuli^18^. Thus, the IOR effect related to the N2 component could occur in visual and audiovisual targets because of the high probability of 80% Go stimuli in this experiment. As mentioned, an IOR effect similar to that of the visual targets should occur in the N2 component in the absence of an integration effect but did not. Thus, multisensory integration is not the only factor. Additionally, IOR was detected only on the P3 component of the audiovisual targets rather than the visual targets, reflecting that IOR has been affected by the target modality, perceptual factors known to influence the cueing effects^45,53^. In other words, the neural processing of the audiovisual IOR is also different from the visual IOR in addition to the influence of multisensory integration factors. Previous studies on the combined regulation of N2 and P3 can account for this difference. Edmund et al.^62^ found that some elderly individuals had a loss of inhibitory control (absence of N2), and the lack of inhibitory control might induce the unnecessary deep evaluation of irrelevant stimuli (presence of P3b). Combined with the current results, the visual targets showed a significant IOR effect on the N2 component and required no more P3 processing. However, we failed to observe the IOR effect associated with the N2 component for the audiovisual targets, leading to P3 processing. As described by Edmund et al., the emergence of the P3 component reflects additional processing. If the time course differences of the IOR effect are simply a function of integration, then the neural processing in the time window without integration should be the same. However, in fact, the N2 component is not similar in these two targets, leading to different P3 processing. Therefore, the neural processing of the two types of IOR were fundamentally different.

Specifically, the neural processing of visual IOR reflects two components of perceptual suppression and reaction inhibition. The former may be related to the suggestion that IOR is associated with changes in perceived quality, which is reflected in the reduced P1 amplitude in extrastriate visual areas^45,63^. The latter may be related to the suggestion that the IOR may be a response criterion marked by N2^64^, representing reluctance to respond to the previously valid location. P1 and N2 were both related to the visual IOR in our study, supporting that the visual IOR may comprise multiple components (attentional and reaction) that act flexibly on different stages of cognitive processing^38,65,66^. Whether the IOR triggers attentional or reaction components has been reported to depend on the response preference determined by the task requirements^36^. For example, a speedy response requirement (reducing the quality of stimulus) may reveal more of the criterion-shift component of IOR, whereas guidance that encourages correctly responding (and emphasises a deeper processing of the stimulus) may reveal more of the attentional component of IOR. In some tasks, such as ours, in which speed and accuracy are equally stressed, both components of IOR may operate within different stages of the time distribution^67^. Thus, the effects of IOR on the response criterion and attention are independent, but they operate cooperatively to achieve the same goal—that is fast and accurate performance.

By contrast, the IOR effect of audiovisual targets was only detected at the P3 stage. P3 component is thought to index aspects of perceived stimulus relevance or context updating^68–70^. As another neural marker of IOR, P3 was increased when a Go stimulus was cued^71^ or was otherwise expected^72^ and usually showed a larger amplitude in the valid locations than in the invalid locations, contrary to the trend of the earlier neural component. Joseph et al.^24^ proposed that P3 has a greater influence on the stimuli of valid locations, indicating that reflexive attention mechanisms cause valid location stimuli to be treated as more salient or potentially significant at higher stages of stimuli evaluation. Additionally, P3 is typically sensitive to infrequent stimuli^73^, suggesting a higher level of attention affecting information processing, perhaps by tagging novel events as having greater potential relevance than other stimuli. Other studies also confirmed that unexpected stimuli increase the P3 component of the wave^19^. These results provided some evidence that the neural processing of the audiovisual IOR in the current study occurs late and contrasts the trend of early neural marks. Combined with the target type after the visual cue in this study, the space specific attention bias of the modality can be explained accordingly. For example, a visual cue on the left might effectively "prime" the visual modality for that side, over other sensory modalities^74,75^. When the cues are visual stimuli, we might expect the subsequent target to be visual as well. When the visual targets are accompanied by the auditory targets, the audiovisual targets may exceed the participants' expectations, and their presence at the valid locations may result in an increase in the P3 amplitude. This point was supported by the evidence of the P3 component from auditory targets. Figures 2f,h and 4f,h show P3 enhancement in the valid locations but not in the invalid locations for both auditory targets and audiovisual targets, rather than for visual targets, and this P3 enhancement was not significant for auditory targets. Thus, the P3 component related to the audiovisual IOR effect was not accidental.

Additionally, we found no visual IOR or audiovisual IOR effects associated with the early N1 component. It is debatable whether the N1 component marks IOR. In the discrimination or Go/NoGo tasks, reductions in the N1 component for valid location compared with those for invalid location trials were associated with IOR. In the detection or localisation task, the absence of N1 modulations was associated with IOR. Differences in the experimental paradigm could be crucial for understanding these discrepant results. Martín-Arévalo et al.^76^ pointed out that previous studies do not provide a consistent association between modulations of the N1 component and the observed behaviour IOR effect; if any association exists, it seems highly dependent on some variables such as the task at hand. In this study, the discrimination task combined with the Go targets/NoGo non-targets setting was adopted. We did not observe the IOR effect of N1 modulation, and the related causes need to be further studied.

To our best knowledge, the present study provides the first electrophysiological evidence for the behaviour IOR difference between visual targets and audiovisual targets. The visual IOR was generated from the P1 and N2 components, while the audiovisual IOR was only derived from the P3 component. Multisensory integration from audiovisual targets causes great effects on audiovisual IOR by offsetting perceptual salience reduced at multiple processing stages. Additionally, the information processing patterns of the visual IOR and the audiovisual IOR are fundamentally different besides the influence of integration. In summary, the difference in early and late processing between the visual IOR and audiovisual IOR implies that the two types of targets may have different inhibitory orientation mechanisms.

## Supplementary Information

Supplementary Information.
