# Supplementary material for: Electrophysiological evidence of different neural processing between visual and audiovisual inhibition of return
Source: Sci Rep. 2021 Apr 13;11:8056. doi: 10.1038/s41598-021-86999-1 (PMC8044137; doi:10.1038/s41598-021-86999-1)
Supplement: Supplementary file 1 — Supplementary Information. [file 41598_2021_86999_MOESM1_ESM.pdf]

Electrophysiological evidence of different neural processing between visual and audiovisual inhibition of return

Xiaoyu Tang[1*], Xueli Wang[1], Xing Peng[2*], Qi Li[3], Chi Zhang[4], Aijun Wang[5*] & Ming Zhang[5*]

[1] School of Psychology, Liaoning Collaborative Innovation Center of Children and Adolescents Healthy Personality Assessment and Cultivation, Liaoning Normal University, Dalian 116029, China.

[2] Institute of Aviation Human Factors and Ergonomics, Civil Aviation Flight University of China, Guanghan 618307, China.

[3] School of Computer Science and Technology, Changchun University of Science and Technology, Changchun, 130022, China.

[4] School of Biomedical Engineering, Faculty of Electronic Information and Electrical Engineering, Dalian University of Technology, Dalian 116024, China.

[5] Department of Psychology, Soochow University, Suzhou 215123, China.

[*] Corresponding author: Xiaoyu Tang, Xing Peng, Aijun Wang, & Ming Zhang

[*] Xiaoyu Tang, School of Psychology, Liaoning Normal University, Dalian 116029, China. Email: tangyu-2006@163.com

[*] Xing Peng, Civil Aviation Flight University of China, Guanghan 618307, China. Email: fypx3688@163.com

[*] Aijun Wang, Department of Psychology, Soochow University, Suzhou 215123, China. Email: psy_waj@126.com

[*] Ming Zhang, Department of Psychology, Soochow University, Suzhou 215123, China. Email: psyzm@suda.edu.cn

In our experiment, motor responses were involved for all the target (Go stimuli) while absent for the non-target (NoGo stimuli). The contribution of movement-related activity to Go/NoGo ERP differences has been debated for many years[1], which usually in the N2 and P3 components of the ERP[2-4]. Thus, we compared the N2 and P3 components of the Go targets with the NoGo non-target to exclude potential motor influence in the further analysis. Mean amplitude data was subjected to a 2 (target type: target and non-target) × 2 (location validity: valid and invalid) × 13 (electrode) three factors repeated measures ANOVA.

Results

Comparison of the auditory IOR effect: (A targets *vs.* A non-targets)

N2 component (220 -290 ms). The N2 waveforms and scalp topographies of the A targets and non-targets are included in Fig. 1 (a), (b), (c), (d). A three-way repeated measures ANOVA on the amplitudes revealed significant main effect of the target type [$F(1, 19) = 6.672$; $p < 0.05$; $\eta_p^2 = 0.260$] and electrode [$F(12, 228) = 11.554$; $p < 0.001$; $\eta_p^2 = 0.378$]. The two-way interaction between the target type and electrode was significant [$F(12, 228) = 10.943$; $p < 0.001$; $\eta_p^2 = 0.365$]. Other main effects or two-way interactions were not significant. The three-way interaction of the target type, location validity and electrode also did not reach significance [$F(12, 228) = 0.532$; $p = 0.893$; $\eta_p^2 = 0.027$].

P3 component (350 -400 ms). The P3 waveforms and scalp topographies of the A targets and non-targets are included in Fig. 1 (e), (f), (g), (h). Significant main effect of the target type [$F(1, 19) = 58.816$; $p < 0.001$; $\eta_p^2 = 0.756$] and electrode [$F(12, 228) =$

23.163; $p < 0.001$; $\eta_p^2 = 0.549$] were observed. The main effect of the location validity [$F(1, 19) = 3.605$; $p = 0.073$; $\eta_p^2 = 0.159$] was marginally significant. The two-way interaction between the target type and electrode was significant [$F(12, 228) = 18.479$; $p < 0.001$; $\eta_p^2 = 0.493$]. Other main effects or the two-way interactions were not found significant. The three-way interaction of the target type, location validity and electrode did not reach significance [$F(12, 228) = 1.361$; $p = 0.186$; $\eta_p^2 = 0.067$].

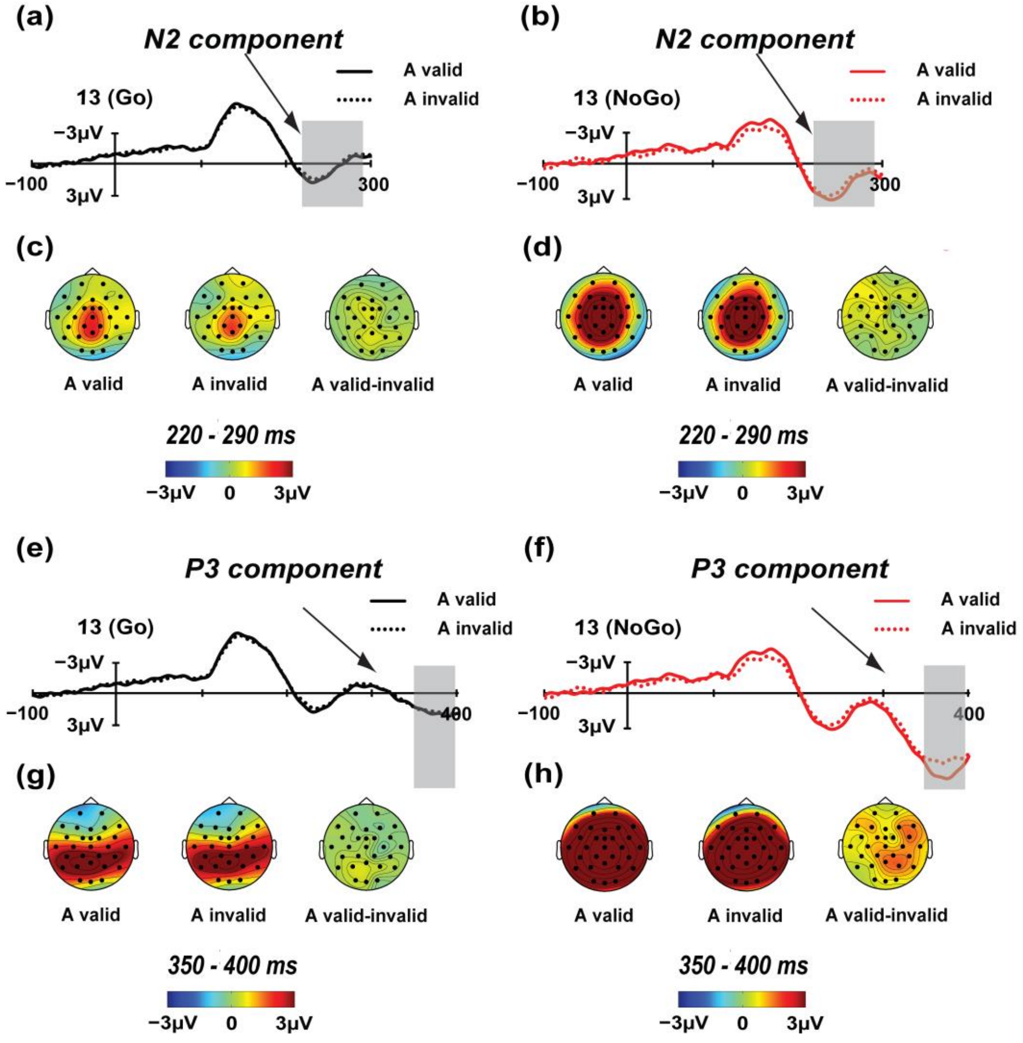

Fig. 1 Direct comparisons of the auditory IOR effect for the time window 220-290 ms and 350-400 ms. The grand average amplitudes elicited by the A targets (black line) and A non-targets (red line) presented in either the valid (solid) or invalid (dotted) locations are shown in panels (a), (b), (e), and (f). The scalp topographies of the N2 and P3 components in the valid and invalid waveforms for targets and non-targets are shown in panels (c), (d), (g), and (h), respectively. Valid-invalid

scalp topographies represent the IOR effect.

Comparison of the visual IOR effect: (V targets *vs.* V non-targets)

N2 component (220 -290 ms). The N2 waveforms and scalp topographies of the V targets and non-targets are included in Fig. 2 (a), (b), (c), (d). Unsurprisingly, we found a significant main effect of the electrode [$F(12, 228) = 16.426$; $p < 0.001$; $\eta_p^2 = 0.464$].

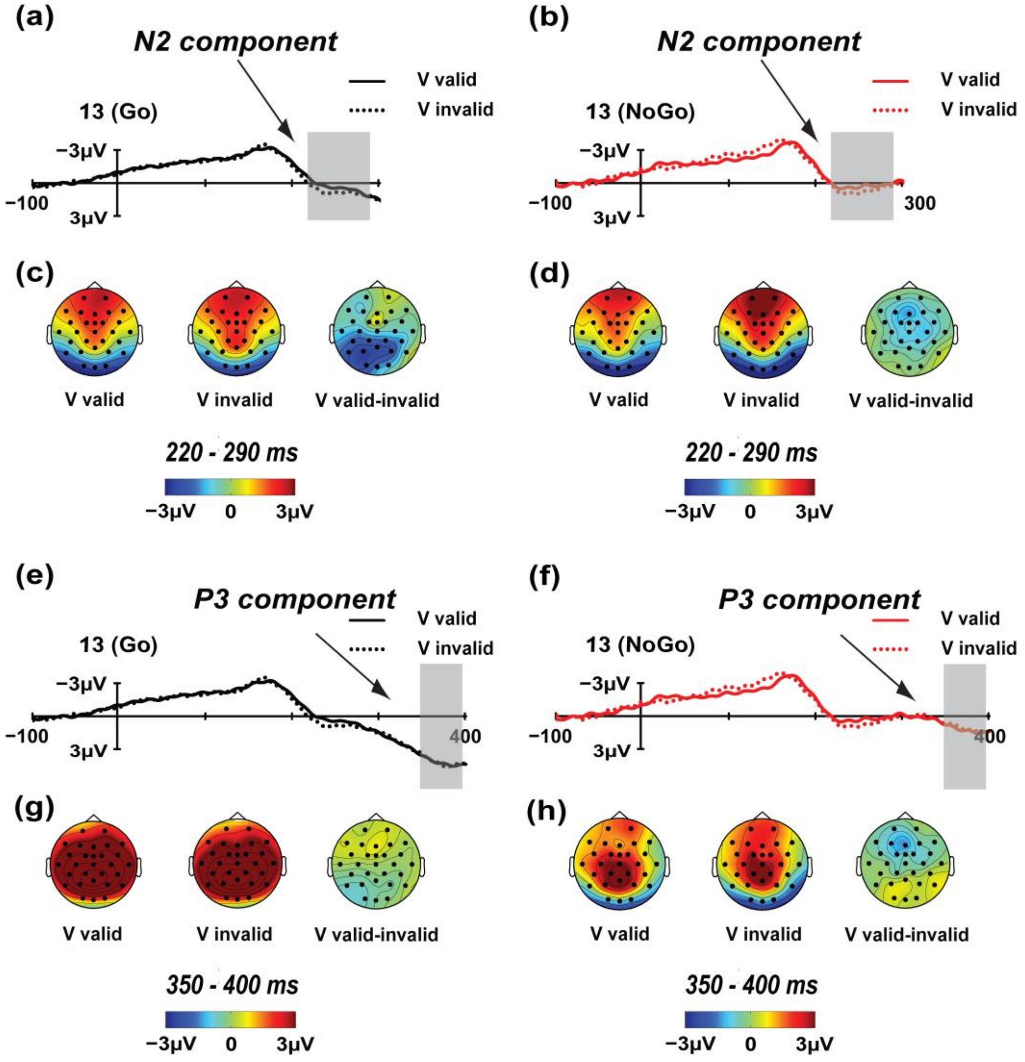

Fig. 2 Direct comparisons of the visual IOR effect for the time window 220-290 ms and 350-400 ms. The grand average amplitudes elicited by the V targets (black line) and V non- targets (red line) presented in either the valid (solid) or invalid (dotted) locations are shown in panels (a), (b), (e), and (f). The scalp topographies of the N2 and P3 components in the valid and invalid waveforms for targets and non-targets were shown in panels (c), (d), (g), and (h), respectively. Valid-invalid

scalp topographies represent the IOR effect.

More importantly, we observed the three-way interaction of the target type, location validity and electrode was significant [$F(12, 228) = 2.100$; $p < 0.05$; $\eta_p^2 = 0.100$]. Further analysis revealed that the significant difference between the valid and invalid locations was found in V targets condition (P3v-i = -0.880 μV, $p < 0.001$; P4v-i = -0.730 μV, $p < 0.05$; P7v-i = -0.692 μV, $p < 0.01$; CP5v-i = -0.599 μV, $p < 0.01$) but not in V non-targets condition. Other main effects or interactions were not found.

P3 component (350 - 400 ms). The P3 waveforms and scalp topographies of the V targets and non-targets are included in Fig. 2 (e), (f), (g), (h). The main effect of the target type [$F(1, 19) = 40.219$; $p < 0.001$; $\eta_p^2 = 0.679$] and electrode [$F(12, 228) = 5.885$; $p < 0.001$; $\eta_p^2 = 0.236$] were significant. The two-way interaction between the target type and electrode [$F(12, 228) = 11.465$; $p < 0.001$; $\eta_p^2 = 0.376$] was significant. The significant three-way interaction of the target type, location validity and electrode was also observed [$F(12, 228) = 2.803$; $p < 0.01$; $\eta_p^2 = 0.129$]. Further analysis revealed that the significant difference between the valid and invalid locations was not found neither in electrodes of V targets condition nor in V non-targets condition. Other main effects or interactions failed to reach significance.

Comparison of the audiovisual IOR effect (AV targets *vs.* AV non-targets)

N2 component (220 - 290 ms). The N2 waveforms and scalp topographies of the AV targets and non-targets are included in Fig. 3 (a), (b), (c), (d). There was a significant main effect of the target type [$F(1, 19) = 10.381$; $p < 0.01$; $\eta_p^2 = 0.353$] and a significant main effect of the electrode [$F(12, 228) = 19.514$; $p < 0.001$; $\eta_p^2 = 0.507$]. The significant

two-way interaction between the target type and electrode [$F(12, 228) = 12.636$; $p < 0.001$; $\eta_p^2 = 0.399$], location validity and electrode were obtained [$F(12, 228) = 2.227$; $p < 0.05$; $\eta_p^2 = 0.105$]. However, the significant three-way interaction of the target type, location validity and electrode was not found [$F(12, 228) = 0.922$; $p = 0.526$; $\eta_p^2 = 0.046$].

P3 component (350 -400 ms). The P3 waveforms and scalp topographies of the AV targets and non-targets are included in Fig. 3 (e), (f), (g), (h). All main effect of the target type [$F(1,19) = 8.681$; $p < 0.01$; $\eta_p^2 = 0.314$], location validity [$F(1,19) = 13.814$; $p < 0.01$; $\eta_p^2 = 0.421$] and electrode [$F(12,228) = 20.408$; $p < 0.001$; $\eta_p^2 = 0.518$] were

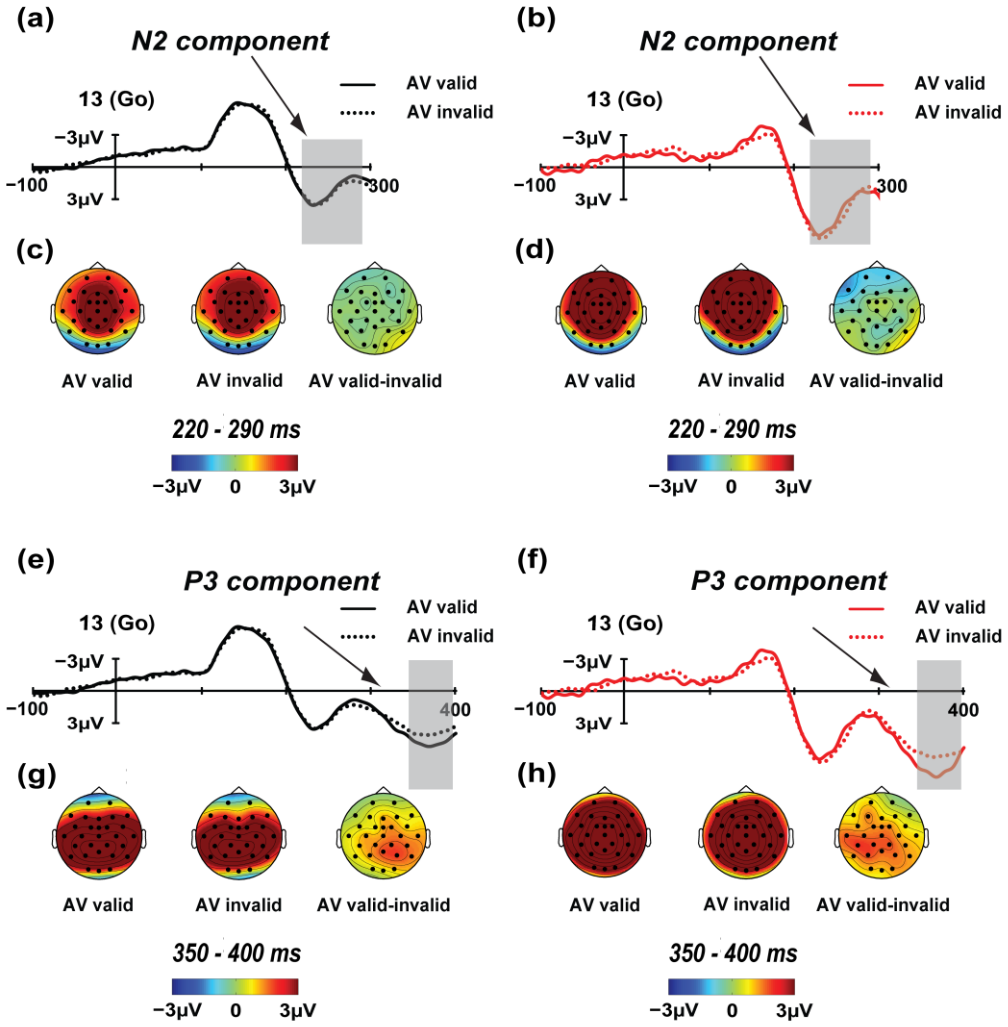

Fig. 3 Direct comparisons of the audiovisual IOR effect for the time window 220-290 ms and 350-400 ms. The grand average amplitudes elicited by the AV targets (black line) and AV non- targets

(red line) presented in either the valid (solid) or invalid (dotted) locations were shown in panel (a), (b), (e), and (f). The scalp topographies of the N2 and P3 components in the valid and invalid waveforms for targets and non-targets were shown in panels (c), (d), (g), and (h), respectively. Valid-invalid scalp topographies represent the IOR effect.

significant. The two-way interaction between the target type and electrode [$F(12, 228) = 13.180$; $p < 0.001$; $\eta_p^2 = 0.410$], location validity and electrode were significant [$F(12, 228) = 2.796$; $p < 0.01$; $\eta_p^2 = 0.128$]. The two-way interaction between the target type and location validity [$F(1, 19) = 0.290$; $p = 0.597$; $\eta_p^2 = 0.015$] and the three-way interaction of the target type, location validity and electrode were not reach significance [$F(12,228) = 0.855$; $p = 0.593$; $\eta_p^2 = 0.043$].

Discussion

The aim of this supplementary analysis was to examine the effect of motor responses on the visual IOR and audiovisual IOR. Mean amplitude analysis of 2 (target type: target, non-target) × 2 (location validity: valid, invalid) × 13 (electrode) revealed that the significant three-way interaction of the target type, location validity and electrode was observed only in visual non-targets. That is, the key press responses was not influence the P3 component of V non-targets, the N2, P3 components of A non-targets and AV non-targets.

It's worth noting that, visual IOR was found in V targets condition rather than in V non-targets condition. This result seems to suggest that the N2 component is influenced by the key press response. However, one issue that remains unresolved in the literature is whether the N2 effects (the difference between Go and NoGo trials) represent cognitive or motor-related aspects of inhibition. Previous found the N2 component of

the ERP did not show a significant difference between Count NoGo and Press NoGo trials[1]. Other researchers have also reported no significant difference between tasks[5,6]. Thus, it appears that the N2 may represent non-motor aspects of inhibition which are equivalent for Press and Count trials. Since the N2 component was increased on NoGo compared to Go trials, leading early researchers to interpret the N2 as a marker of response inhibition[7]. However, no IOR effect relate to N2 was found in the current visual non-target condition (NoGo). Such evidence indicated that current results cannot be explained by a motor inhibitory of N2. In other words, the N2 difference between targets and non-targets was not due to key press actions. Another studies also revealed that N2 reflects some other mental processing. For example, the N2 was sensitive to the Go/NoGo stimulus probability[8], task instructions or the changes in response criteria[3]. The absence of N2 effect in current non-target trials may be related to these factors, further study should to be considering.

References

1. Smith, J. L., Jamadar, S., Provost, A. L. & Michie, P. T. Motor and non-motor inhibition in the Go/NoGo task: An ERP and fMRI study. *Int J Psychophysiol* 87,244-253, doi:https://doi.org/10.1016/j.ijpsycho.2012.07.185 (2013).
2. Tian, Y. & Yao, D. A study on the neural mechanism of inhibition of return by the event-related potential in the Go/NoGo task. *Biol Psychol* 79, 171-178, doi:https://doi.org/10.1016/j.biopsycho.2008.04.006 (2008).
3. Prime, D. J. & Jolicoeur, P. Response-selection conflict contributes to inhibitiono f return. *J Cogn Neurosci* 21, 991-999, doi:https://doi.org/10.1162/jocn.2009.21105 (2009).

4. Smith, J. L., Smith, E. A., Provost, A. L. & Heathcote, A. Sequence effects support the conflict theory of N2 and P3 in the Go/NoGo task. *Int J Psychophysiol* 75,217-226, doi:https://doi.org/10.1016/j.ijpsycho.2009.11.002 (2010).
5. Smith, J. L., Johnstone, S. J. & Barry, R. J. Movement-related potentials in the Go/NoGo task: The P3 reflects both cognitive and motor inhibition. *Clin Neurophysiol* 119, 704-714, doi:https://doi.org/10.1016/j.clinph.2007.11.042 (2008).
6. Nakata, H. *et al.* Effects of a go/nogo task on event-related potentials following somatosensory stimulation. *Clin Neurophysiol* 115, 361-368, doi:https://doi.org/10.1016/j.clinph.2003.09.013 (2004).
7. Kok, A. Effects of degradation of visual stimuli on components of the event-related potential (ERP) in go/nogo reaction tasks. *Biol Psychol* 23, 21-38, doi:https://doi.org/10.1016/0301-0511(86)90087-6 (1986).
8. Nieuwenhuis, S., Yeung, N., van den Wildenberg, W. & Ridderinkhof, K. R. Electrophysiological correlates of anterior cingulate function in a go/no-go task: Effects of response conflict and trial type frequency. *Cogn Affect Behav Neurosci* 3, 17-26, doi:https://doi.org/10.3758/CABN.3.1.17 (2003).
